# Commensal Microbes Affect Host Humoral Immunity to *Bordetella pertussis* Infection

**DOI:** 10.1128/IAI.00421-19

**Published:** 2019-09-19

**Authors:** Youyi Zhang, Zihan Ran, Miaomiao Tian, Yang Zhou, Jingcheng Yang, Juan Yin, Daru Lu, Rui Li, Jiang Zhong

**Affiliations:** a Department of Microbiology and Microbial Engineering, School of Life Sciences, Fudan University, Shanghai, People’s Republic of China; b Key Laboratory of Birth Defects and Reproductive Health of National Health and Family Planning Commission, Chongqing Key Laboratory of Birth Defects and Reproductive Health, Chongqing Population and Family Planning, Science and Technology Research Institute, Chongqing, People’s Republic of China; c Inspection and Quarantine Department, The College of Medical Technology, Shanghai University of Medicine & Health Sciences, Shanghai, People’s Republic of China; d State Key Laboratory of Genetic Engineering, School of Life Sciences and Human Phenome Institute, Fudan University, Shanghai, People’s Republic of China; University of California San Diego School of Medicine

**Keywords:** *Bordetella pertussis*, humoral immunity, PD-1, microbiota

## Abstract

As important players in the host defense system, commensal microbes and the microbiota influence multiple aspects of host physiology. Bordetella pertussis infection is highly contagious among humans. However, the roles of the microbiota in B. pertussis pathogenesis are poorly understood. Here, we show that antibiotic-mediated depletion of the microbiota results in increased susceptibility to B. pertussis infection during the early stage. The increased susceptibility was associated with a marked impairment of the systemic IgG, IgG2a, and IgG1 antibody responses to B. pertussis infection after antibiotic treatment. Furthermore, the microbiota impacted the short-lived plasma cell responses as well as the recall responses of memory B cells to B. pertussis infection. Finally, we found that the dysbiosis caused by antibiotic treatment affects CD4^+^ T cell generation and PD-1 expression on CD4^+^ T cells and thereby perturbs plasma cell differentiation. Our results have revealed the importance of commensal microbes in modulating host immune responses to B. pertussis infection and support the possibility of controlling the severity of B. pertussis infection in humans by manipulating the microbiota.

## Abstract (precis)

As important players in the host defense system, commensal microbes and the microbiota influence multiple aspects of host physiology. Bordetella pertussis infection is highly contagious among humans. However, the roles of the microbiota in B. pertussis pathogenesis are poorly understood. Here, we show that antibiotic-mediated depletion of the microbiota results in increased susceptibility to B. pertussis infection during the early stage.

## INTRODUCTION

Pertussis, also known as whooping cough, is a highly contagious respiratory disease that is transmitted directly from human to human (1). Bordetella pertussis, a strict human pathogen, is the etiological agent of whooping cough. Despite the availability and intensive use of efficacious vaccines for several decades, pertussis has not been eradicated and has recently reemerged as a major public health threat (2, 3). In fact, B. pertussis strains keep circulating in many countries with high vaccine coverage, and reports of an increasing incidence of B. pertussis infection worldwide have been accumulating for the past 20 years (3–5). The reemergence of pertussis cases has partially been attributed to the waning immunity conferred by current acellular pertussis vaccines, medical advancements that have allowed more effective diagnosis and reporting of pertussis cases, the asymptomatic transmission of B. pertussis from individuals vaccinated with the acellular pertussis vaccine, evolving variant strains of circulating B. pertussis strains against which humans are less protected by the vaccine, and a decrease in vaccine coverage, which has compromised herd/community immunity (6, 7).

Several virulence factors, such as pertussis toxin (PT), adenylate cyclase toxin (AC), dermonecrotic toxin (DNT), and tracheal cytotoxin (TCT), have been shown to play pivotal roles in B. pertussis pathogenesis (1, 2, 8). Protection against B. pertussis infection is mediated by the innate and adaptive immune responses, and complete bacterial clearance requires both cell-mediated and humoral responses (9). However, combined data from clinical and animal studies showed that B. pertussis-specific antibodies are important protective immune reactants against B. pertussis infection. Antibodies against major B. pertussis virulence factors are capable of preventing bacterial colonization by blocking adherence of the bacteria to human epithelial cells (10). The importance of B cells and antibodies in the protection against B. pertussis infection has also been demonstrated in studies using Ig^−/−^ knockout mice (11). In addition, passive transfer experiments in animals showed that passively transferred anti-B. pertussis antibodies protected naive mice and piglets against B. pertussis challenge (12, 13). Long-term infection by B. pertussis is mainly caused by the ability of B. pertussis to interfere with the host’s innate and adaptive immune systems. Despite great advances in our understanding of B. pertussis virulence factors, the interplay between the pathogen and the host, especially the host microecology, still remains poorly understood.

Commensal microbes can influence multiple aspects of host physiology (14), including host susceptibility to numerous diseases (15–19). Indeed, the intestinal microbiota has emerged as a positive player in the host defense system, supporting mucosal immunity and potentially modulating systemic immunity (20–23). The effect of the gut microbiota on the immune responses in distal mucosal sites and its impact on the outcome of respiratory infections have recently been posed. In this regard, some studies have shown that the gut microbiota plays a crucial role in the response to bacterial and viral respiratory infections (24–28). Recently, several studies have shown that dysbiosis of the microbiota can cause long-lived immunological scarring, with profound effects on host immunity (29–31). Ruiz et al. showed that a single early-life macrolide course can alter the microbiota and modulate host immune phenotypes and that these effects persist long after the antibiotic exposure has ceased (29). In addition, they showed that early-life antibiotic exposure has lasting and transferable effects on the microbial community network topology (29).

One aspect of B. pertussis infection that remains largely unexplored is the role of the host microbiota in B. pertussis pathogenesis. To date, only one study has focused on the role of the nasal resident microbiota in bacterial competition with initial colonization and host selection during B. pertussis infection (32), where the authors showed that the removal of resident microorganisms from the nasal cavity allowed B. pertussis to efficiently colonize the murine nasal cavity and that the reintroduction of a single nasal cavity bacterial species was sufficient to block B. pertussis colonization in mice. However, the exact mechanisms by which commensal microbes prevent B. pertussis colonization are still unclear.

In the present study, we investigated the impact of commensal microbes on host immune responses during B. pertussis infection. We found that the microbiota dysbiosis caused by antibiotic treatment increases the pulmonary bacterial burden during early infection and contributes significantly to impaired host primary and secondary immune responses through the regulation of CD4 helper T cell generation and PD-1 expression.

## RESULTS

### Broad-spectrum antibiotic treatment leads to dramatic changes in the gut microbiota.

**FIG 1:**
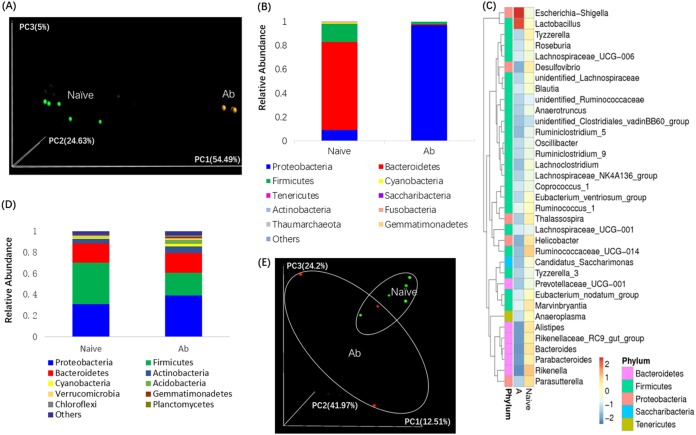
Effect of broad-spectrum antibiotic treatment on microbial profile in mouse feces and lungs. (A) 3D-PCoA score plot of the gut microbiota in naive mice and mice treated with antibiotics, based on weighted UniFrac metrics. Each dot represents an individual mouse. (B) Phylogenetic profile of bacterial phyla in the feces of mice treated or not treated with antibiotics. Stacked bar charts show the 10 main phyla, identified on the basis of their relative abundance in antibiotic-treated or non-antibiotic-treated mice 3 days after the cessation of antibiotic treatment. (C) Heat map demonstrating the relative abundance of the dominant bacterial phyla in the feces of naive mice and mice treated with antibiotics. (D) Phylogenetic profile of bacterial phyla in the lungs of mice treated or not treated with antibiotics. Stacked bar charts show the 10 main phyla, identified on the basis of their relative abundance in antibiotic-treated or non-antibiotic-treated mice 3 days after the cessation of antibiotic treatment. (E) 3D-PCoA score plot of the lung microbiota in naive mice and mice treated with antibiotics, based on weighted UniFrac metrics. Each dot represents an individual mouse. Ab and A, antibiotic-treated mice.

To determine the extent to which the intestinal microbiota provides resistance against nasal infection with B. pertussis, BALB/c mice were treated with a cocktail of four antibiotics for 3 weeks, followed by the nasal administration of 5 × 10^6^ CFU of B. pertussis BPMM in 20 μl. As demonstrated in a three-dimensional (3D) principal-component analysis (PCoA) score plot, broad-spectrum antibiotic treatment resulted in marked changes in the composition of the gut bacteria (beta diversity, *P ≤ *0.001) (Fig. 1A). Further phylum-level analysis identified several layers of bacterial taxa that differed in relative abundance after antibiotic treatment (Fig. 1B and C). The major phyla present in the mouse intestinal microbiota were *Bacteroidetes*, *Firmicutes*, and *Proteobacteria*; however, after antibiotic treatment, we observed a marked reduction in the *Bacteroidetes* (72.58%) and *Firmicutes* (13.38%) and a dramatic expansion of the *Proteobacteria* (87.65%). Nevertheless, oral antibiotic treatment did not seem to affect the bacterial composition in the lungs, as demonstrated by PCoA (Fig. 1D) and phylum-level bacterial abundance analysis (Fig. 1E) (beta diversity, *P* = 0.331).

### The host microbiota is necessary for early inhibition of B. pertussis infection.

**FIG 2:**
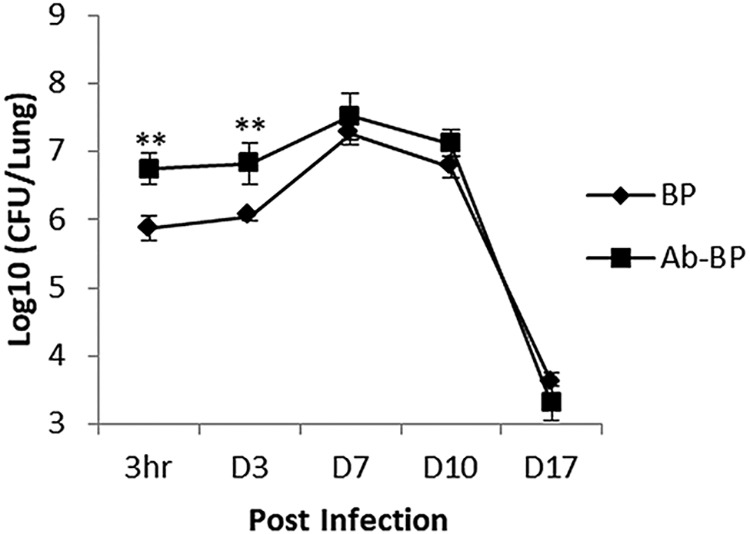
The microbiota is necessary for early inhibition of B. pertussis colonization in the lungs. The lung colonization profiles of B. pertussis strain BPMM (BP) in mice treated or not treated with antibiotics (Ab) were measured. BALB/c mice were infected intranasally with 5 × 10^6^ CFU of BPMM. The lungs were harvested at the indicated time points and homogenized. Appropriate dilutions of the lung homogenates were plated onto blood agar plates, and the number of CFU was counted after 4 days of incubation at 37°C. Four mice per group per time point were assessed individually. Results are expressed as the mean of the log_10_ number of CFU per lung ± standard deviations (SD) calculated for each mouse. **, *P* ≤ 0.01 compared with the values obtained between two groups at the same time points. D, day.

Upon intranasal inoculation, strain BPMM displayed a slight peak of multiplication at day 7, followed by a progressive clearance of the bacteria from the lungs (Fig. 2). However, in spite of the comparable colonization profiles at later time points, antibiotic treatment markedly increased the magnitude of B. pertussis carriage in the lungs at 3 h and 3 days postinfection (Fig. 2). Thus, our data show that an intact, balanced microbiota inhibits the early colonization of B. pertussis in the lungs and that the microbiota dysbiosis caused by antibiotic administration to mice before infection results in increased susceptibility to B. pertussis infection at early stages.

### The host microbiota affects early systemic antibody responses to B. pertussis.

**FIG 3:**
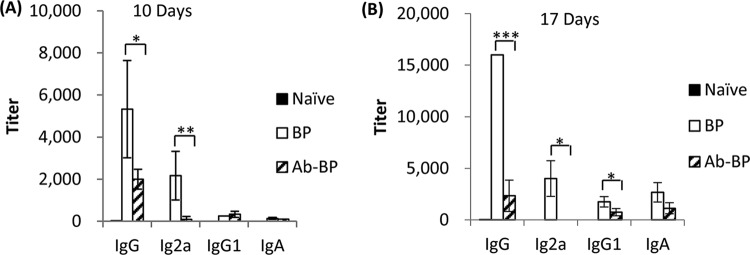
The host microbiota is necessary for early systemic antibody responses to B. pertussis. The systemic anti-B. pertussis antibody responses in antibiotic-treated mice and nontreated naive mice nasally infected with BPMM were analyzed. Groups of 4 adult BALB/c mice were i.n. infected with 5 × 10^6^ CFU of BPMM. Sera were collected at 10 and 17 days postinfection. Systemic anti-BPMM total IgG, IgG2a, IgG1, and IgA titers in serially diluted individual serum samples were measured by ELISA using BPMM whole-cell lysate as the coating antigen. The results are representative of those from three independent experiments. *, *P* ≤ 0.05; **, *P* ≤ 0.01; ***, *P* ≤ 0.001.

As systemic antibody responses to B. pertussis play essential roles against B. pertussis infections, we next investigated the impact of the host microbiota on the early rapid induction of antibody responses following B. pertussis infection. We found that B. pertussis-specific IgG and IgG2a titers were significantly reduced in the antibiotic-treated mice at 10 and 17 days after infection (Fig. 3A and B). At 17 days postinfection, B. pertussis-specific IgG1 titers were also remarkably different between antibiotic-treated mice and their littermate control mice. Importantly, the IgG2a/IgG1 ratio was markedly lower in antibiotic-treated mice infected with B. pertussis than in antibiotic-treated mice not treated with antibiotics, which is indicative of a shift from a Th1-oriented immune response to a Th2-oriented immune response. No significant difference in B. pertussis IgA titers was observed between mice treated with antibiotics and mice not treated with antibiotics at both 10 days and 17 days postinfection. In addition, comparable levels of serum IgM were found between antibiotic-pretreated mice and nontreated control mice, indicating that the reduced production of IgG antibodies is not a result of an impaired competence of B cells for a class switch recombination from IgM to other isotypes (see Fig. S1 in the supplemental material). Taken together, our results demonstrate that the microbiota affects host antibody responses both quantitatively and qualitatively.

### The microbiota impacts the short-lived PC response after B. pertussis infection.

**FIG 4:**
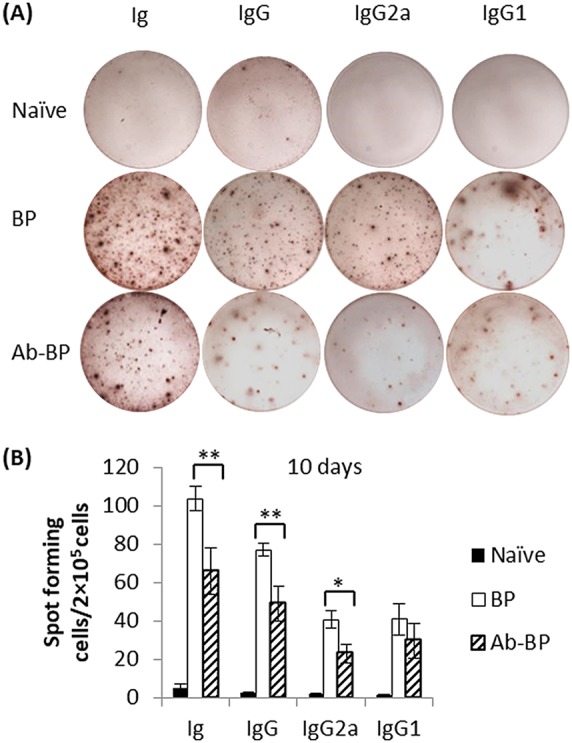
Impaired short-lived plasma cell response to B. pertussis infection in antibiotic-treated mice. B. pertussis-specific antibody-secreting cells (ASCs) in the spleens of mice treated or not treated with antibiotics were measured at 10 days postinfection by an ELISPOT assay. Representative spot formations (A) and total frequencies, expressed as the mean ± SD (B), are presented. Data are representative of those from two independent experiments. *, *P* ≤ 0.05; **, *P* ≤ 0.01.

To investigate the cellular mechanisms underlying the impact of the host microbiota, particularly during the early phase of the humoral immune response, we examined the B cells in the spleens of antibiotic-treated mice 10 days after B. pertussis infection. B cell enzyme-linked immunosorbent spot (ELISPOT) assays were carried out to measure the frequency of B. pertussis-specific short-lived plasma cells (PCs). Consistent with the amount of B. pertussis-specific antibodies detected in the serum, there was a substantial reduction in the frequency of B. pertussis-specific antibody-secreting cells (ASCs) in the spleens of antibiotic-treated mice 10 days after infection (Fig. 4A and B). In mice treated with antibiotics, the reduction in antigen-specific ASCs remarkably reflected the reduction of B. pertussis-specific antibody concentrations in the serum compared to those in the serum of their nontreated counterparts. Thus, our data suggest that the majority of B. pertussis-induced total Ig, IgG, IgG2a, and IgG1 antibodies detected during the first 10 days after infection are produced by plasmablasts or short-lived plasma cells and that the microbiota was critical for the induction of these cell types.

### Impact of the host microbiota on the colonization and recall response of memory B cells against secondary B. pertussis infection.

**FIG 5:**
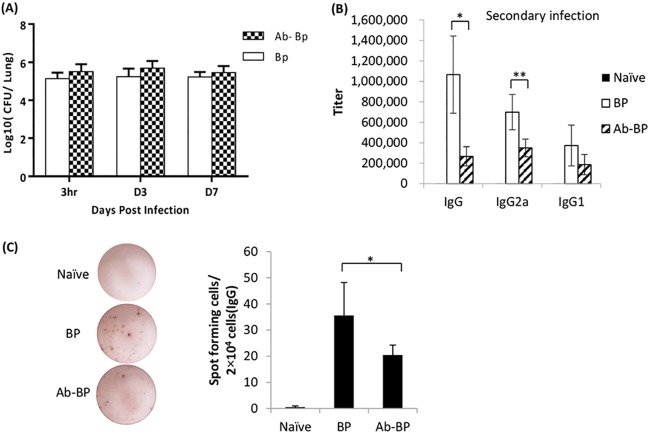
Microbiota dysbiosis caused by antibiotic treatment leads to an impaired recall response of memory B cells against B. pertussis infection. (A) Bacterial carriage in the lungs was measured at 3 h, 3 days, and 7 days after infection and compared. (B and C) B. pertussis-specific IgG, IgG2a, and IgG1 titers (B) as well as the frequencies of B. pertussis-specific IgG ASCs in the spleens (C) were measured in mice treated or not treated with antibiotics and infected twice with B. pertussis. Analysis was performed 14 days after secondary B. pertussis infection. Data are expressed as the mean ± SD. *, *P* ≤ 0.05; **, *P* ≤ 0.01.

To further determine the degree to which the microbiota impacts the bacterial colonization ability and host antibody responses, we investigated the colonization profile and antibody responses to a secondary B. pertussis infection in mice treated with antibiotics. Mice were given a second administration of B. pertussis using the same dose and route used for the primary administration. No antibiotics were used prior to the second B. pertussis challenge. As can be seen from Fig. 5A, the microbiota dysbiosis caused by antibiotic treatment did not significantly affect the colonization ability of secondary B. pertussis infection. However, to our surprise, the magnitude of the secondary antibody response was substantially lower in antibiotic-treated mice than in their littermate control mice. Both B. pertussis-specific IgG and IgG2a concentrations were significantly lower in antibiotic-treated, B. pertussis-infected mice than in mice that did not receive antibiotic pretreatment (Fig. 5B). No significant differences in B. pertussis-specific IgG1, IgA, and IgM titers were found between the sera of mice treated and the sera of mice not treated with antibiotics (Fig. 5B and Fig. S2). These results suggest that the microbiota plays an important role in the differentiation or function of memory B cells following infection with B. pertussis. Furthermore, the IgG2a-oriented immune responses observed in B. pertussis-infected mice without antibiotic pretreatment remained greatly impaired in mice infected with B. pertussis and treated with antibiotics.

To determine whether antigen-specific ASCs arising from the recall response of memory B cells are influenced by the microbiota, antibiotic-treated mice were given a boost administration, as described above. We found fewer IgG-secreting cells in antibiotic-treated mice than in mice not treated with antibiotics at day 14 after secondary infection (Fig. 5C). A pairwise analysis between the corresponding serum samples tested by enzyme-linked immunosorbent assay (ELISA) and the B cell ELISPOT assay revealed a positive correlation between the frequency of ASCs and the magnitude of B. pertussis-specific IgG antibodies in the serum (Fig. 5C). Our data suggest that the magnitude of ASC induction following secondary infection with B. pertussis is also critically dependent on the host microbiota.

### B. pertussis-specific antibodies restored the colonization resistance to B. pertussis in antibiotic-treated mice.

**FIG 6:**
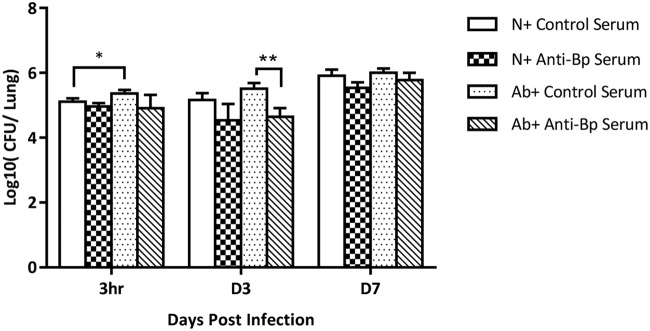
B. pertussis-specific antibodies restored the colonization resistance to B. pertussis in antibiotic-treated mice. Antibiotic-treated mice and naive mice were i.p. injected with 200 μl of naive or high-titer anti-BPZE1 immune serum before B. pertussis infection. Lung colonization of B. pertussis was measured 3 h, 3 days, and 7 days after infection and compared. Data are expressed as the mean ± SD. *, *P* ≤ 0.05; **, *P* ≤ 0.01.

To further investigate whether the increased colonization of B. pertussis in antibiotic-treated mice was indeed driven by an impaired antibody response, we carried out a pathogen-specific antibody passive transfer experiment. Control mice or antibiotic-treated mice were intraperitoneally (i.p.) injected with 200 μl of naive or anti-BPZE1 immune serum. As can be seen in Fig. 6 for naive and antibiotic-treated mice receiving control serum, antibiotic treatment markedly increased the magnitude of B. pertussis carriage in the lungs at 3 h postinfection. However, in naive and antibiotic-treated mice receiving anti-B. pertussis serum, no significant differences in B. pertussis carriage in the lungs were found. Thus, our data show that B. pertussis-specific antibodies successfully restored the colonization resistance to B. pertussis in antibiotic-treated mice.

### The microbiota affects CD4^+^ T cell generation and PD-1 expression in T cells.

**FIG 7:**
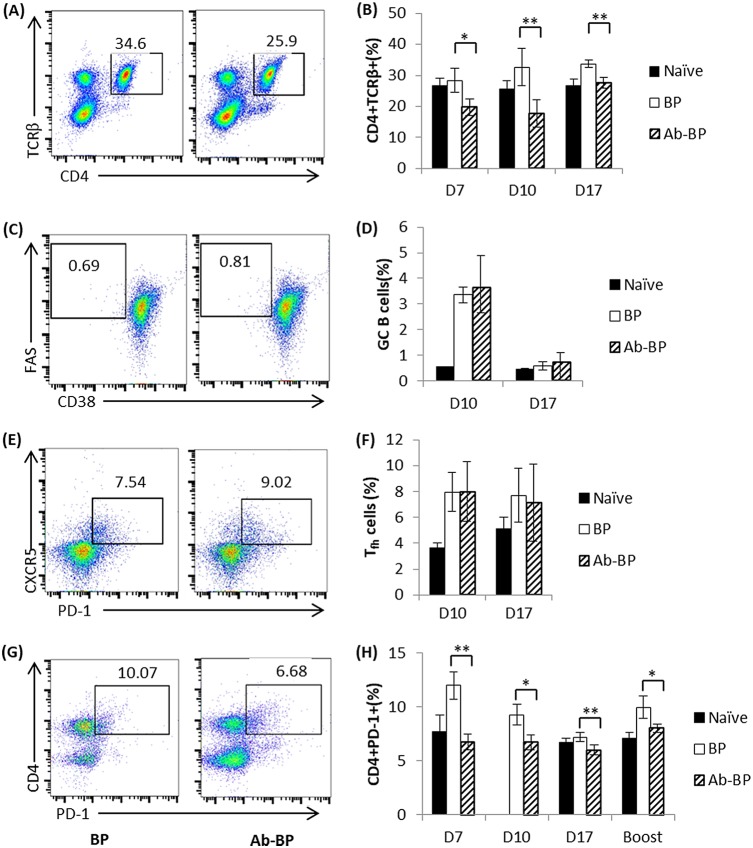
Impaired CD4^+^ T cell generation and PD-1 expression in T cells of mice treated with antibiotics infected with B. pertussis. (B, D, F, and H) Naive mice or mice treated with antibiotics were infected with BPMM, and the frequencies of CD4^+^ T cells (B), GC B cells (D), T_fh_ cells (F), and PD-1 expression on CD4^+^ T cells in the mouse spleens (H) were measured at the indicated time points following infection. Four mice per group per time point were analyzed individually. (A, C, E and G) Representative flow cytometry analyses. Data are expressed as the mean ± SD. *, *P* ≤ 0.05; **, *P* ≤ 0.01.

Since microbiota dysbiosis by antibiotic treatment not only resulted in a drastic impairment of systemic antibody responses to B. pertussis infection but also caused a substantial alteration in antibody isotypes, we first examined the frequency of CD4^+^ helper T cells in the spleens of antibiotic-treated mice and the non-antibiotic-treated control mice. We found a marked reduction in the number of CD4^+^ T helper cells (CD4^+^ T cell receptor β [TCRβ]-positive [TCRβ^+^]) in mice treated with antibiotics compared with that in the nontreated control mice (Fig. 7A and B). Next, germinal center (GC) B cell and T follicular helper (T_fh_) cell generation in the spleens of mice treated or not treated with antibiotics was further investigated. The gating strategies used for the identification of GC B (CD19^+^ CD38-Fas^+^) and T_fh_ (CD4^+^ TCRβ^+^ PD-1^+^ CXCR5^+^) cells are shown in Fig. S4. To our surprise, the antibiotic-treated group displayed populations of GC B and T_fh_ cells comparable to those in the non-antibiotic-treated group (Fig. 7C to F). These results indicate that, rather than influencing GC B and T_fh_ cell generation, antibiotic treatment affected the optimal T cell-dependent antibody responses during the generation of adaptive immune responses.

GC B cells differentiate into antibody-secreting PCs during the T cell-dependent antibody response (33, 34). Interruptions in the T cell-B cell cognate interactions have been shown to affect PC differentiation and antibody generation (33–35). Especially, several studies have shown that PD-1 on T cells can regulate selection and survival in the germinal center and affect the quantity and quality of long-lived PCs (36, 37). In the present study, a substantial reduction in the frequency of B. pertussis-specific ASCs was detected 10 days after infection in the antibiotic-treated mice compared with the non-antibiotic-treated control mice (Fig. 4A and B). The same phenomenon was also observed for secondary infection (Fig. 5B and C). Hence, we continued to analyze the expression of PD-1 on CD4^+^ T cells in the spleens through multiple time points in mice treated or not treated with antibiotics after primary and secondary B. pertussis infection. Interestingly, we consistently found PD-1 expression to be significantly lower on CD4^+^ T cells in the spleens of mice treated with antibiotics than in those of the nontreated control mice (Fig. 7G and H). Thus, taken together, our data suggest that the dysbiosis caused by antibiotic treatment affects the expression of PD-1 on CD4^+^ T cells and thereby perturbs PC differentiation.

## DISCUSSION

The study presented here reveals four major findings relevant to our understanding of systemic and mucosal humoral responses in B. pertussis infection: (i) dysbiosis of the microbiota by antibiotic treatment results in increased susceptibility to B. pertussis infection at early stages; (ii) the systemic primary and secondary IgG, IgG2a, and IgG1 antibody responses to B. pertussis infections are substantially impaired by antibiotic treatment; (iii) the host microbiota impacts the short-lived plasma cell response and recall response of memory B cells to B. pertussis infection; and (iv) microbiota dysbiosis by antibiotic treatment significantly affects CD4^+^ T cell generation and PD-1 expression on T cells during B. pertussis infection and thereby perturbs plasma cell function. Our study reveals that significant impairment of humoral responses may have contributed to the increased susceptibility to B. pertussis infection. Our study underscores the concept that dysbiosis of the microbiota can lead to long-lived immunological scarring, with profound effects on host immunity to subsequent infections. Our work also facilitates our understanding of B. pertussis pathogenesis and its interaction with the host during infection.

In the present study, we observed that treatment with a broad-spectrum antibiotic cocktail results in significant marked changes in the composition of gut bacteria at the time of infection. However, the influence of antibiotic administration on microbiota is long-lived and transferable. Jernberg et al. showed that clindamycin treatment affected the *Bacteroides* bacteria in the gut for up to 2 years after treatment had finished (38). Similarly, three individuals with dyspepsia treated for 1 week with a combination of metronidazole, clarithromycin, and omeprazole had a shift in their microbiota state that persisted for up to 4 years without additional antibiotic treatment (39). In addition, Dethlefsen and Relman showed that ciprofloxacin treatment led to profound and rapid changes in the gut microbiota (40). Communities began to return to their initial state by 1 week after the end of each course, but the return was often incomplete (40). Surprisingly, Gonzalez-Perez and colleagues showed that maternal antibiotic treatment/treated (MAT) during pregnancy and lactation resulted in profound alterations in the composition of the gastrointestinal tract microbiota in mothers and infants, conferring increased susceptibility to death following a systemic viral infection (41). Thus, antibiotic treatment has profound and far-reaching influences on the host microbiota. The composition of the gut microbiota was stabilized by the end of the experiment but was altered from its initial state, leading to long-term microbiota dysbiosis.

Substantial research has shown that the microbiota, especially the gut microbiota, is a key player in the modulation of host intestinal immune responses (42–45). However, the capacity of the microbiota to influence host immunity may be more pervasive and far-reaching. The effect of the gut microbiota on the immune responses to infections in extragastrointestinal sites has recently been exposed (15, 26, 28). Moreover, a number of recent studies have demonstrated that the commensal microbiota enhances the initial innate response to lung infection by bacteria (24, 27, 46, 47). In the present study, we demonstrated that the murine gut microbiota influences host humoral immune responses to intranasal B. pertussis infection, with most B. pertussis-specific Ig production being markedly impaired and the pulmonary bacterial burden being greatly increased by antibiotic treatment. In addition, as primary immune responses play vital roles in the induction of secondary immune responses, a surprising aspect of our study was the long-term effect of gut microbiota dysbiosis on host immune responses to secondary infections. It is probably a result of the inefficient induction of primary immune responses due to antibiotic treatment. We further demonstrated that an intact microbiota is critical for the induction of short-lived PC responses and that microbiota dysbiosis also impairs recall memory B cells. These results are consistent with previous observations that fewer long-lived plasma cells (PCs) are induced in the absence of microbiota (48). Moreover, an antibody passive transfer experiment showed that B. pertussis-specific antibodies could successfully restore colonization resistance to B. pertussis in antibiotic-treated mice. Our data demonstrate that the importance of the gut microbiota in host defense against respiratory infection is probably mediated through the regulation of host humoral immune responses against infection.

On the other hand, our study supports the idea that the gut microbiota plays a significant role in the host response to vaccines and that antibiotic treatment before vaccination may greatly affect the protective efficacy of vaccines that rely mostly on antibodies. As mentioned above, studies have shown that the influence of antibiotic administration on microbiota is far-reaching. Antibiotic treatment can move the microbiota to alternative stable states. The composition of the gut microbiota stabilized by the end of the experiment but was altered from its initial state. The diversity of the microbiota may return to normal in a few weeks; however, some bacterial taxa were eliminated for a period of 6 months (49). The results from these studies may help guide treatment strategies, such as the selection of antibiotics that are less likely to have long-term effects on the commensal microbiota and the use of attempts to recover or optimize the microbiota before vaccination.

The cellular and molecular mechanisms through which the microbiota maintains and modulates immune responses in mucosal sites are still poorly understood. In this study, we revealed that microbiota dysbiosis by antibiotic treatment affected CD4^+^ T cell generation and the expression of PD-1 on these cells, leading to the impairment of PC differentiation and antibody responses. Our data demonstrate that not only the production of immune cells but also the functioning of these cells is controlled by the microbiota. Although no difference in T_fh_ cells was observed between mice treated with antibiotics and mice not treated with antibiotics, the decreased expression of PD-1 expression may result in interruptions of B cell-T cell cognate interactions, which ultimately affect GC formation and PC differentiation (33, 34). Furthermore, it was reported that the PD-1 pathway inhibits the function of follicular regulatory T cells (T_fr_), and PD-1-deficient T_fr_ cells have an enhanced ability to suppress antibody production (50). Nevertheless, the precise cellular and molecular mechanisms underlying the observed reduction of CD4^+^ T cell generation and PD-1 expression specific to respiratory microbial antigens are unclear at present and are most likely multifactorial. It has previously been shown that the gut microbiome provides signals to monocytes/macrophages and primes those cells to respond to and help control systemic lymphocytic choriomeningitis virus infections (28). Kim et al. demonstrated that the short-chain fatty acids (SCFAs) produced by the gut microbiota support host antibody responses during steady state and infection by regulating gene expression and B cell differentiation (51). Whether the gut microbiome modulates host immunity to B. pertussis infection through similar or different effects on the host immune system warrants further investigation.

The host microbiota is critical to protecting against invading pathogens. Information on the effects of resident microorganisms on the initial stages of B. pertussis infection is still lacking. The only study of this, one by Weyrich et al., showed that the nasal resident microbiota competes with B. pertussis against its initial colonization during infection (32). However, there are substantial differences between their study and ours. Weyrich and colleagues (32) directly inoculated antibiotics into the mouse nasal cavity, which directly influences nasal microorganisms, whereas in our study, antibiotics were given orally, which greatly influenced the microbiota in the gut instead of that in the nasal cavities. On the other hand, in our study, B. pertussis infection was performed 3 days after the cessation of oral antibiotic treatment. At the time of B. pertussis infection, it seems that the microbiota had recovered to its original state, based on our analysis of the bacterial composition in the lungs of mice treated or not treated with antibiotics (Fig. 1). What is more, although our data (Fig. 2) showed that the effect of the microbiota in protecting against B. pertussis colonization was limited only to the first 3 days, it is still of great importance because the earliest events in the colonization of a host are critical aspects of disease transmission, spread, and pathogenesis. Further, microbiota dysbiosis had a significant and far-reaching influence on host humoral immune responses against B. pertussis infection. Our data showed that not only humoral immune responses to primary B. pertussis infection but also those to secondary B. pertussis infection were greatly suppressed.

Last but not the least, the sequencing results for the gut and lung microbiota showed that the gut microbiota was the most affected by oral antibiotic treatment. At the phylum level, both the *Bacteroidetes* and *Firmicutes* decreased markedly after antibiotic treatment, whereas the *Proteobacteria* increased dramatically after antibiotic treatment (Fig. 1B). Indeed, several studies have reported an increased abundance of *Proteobacteria* in the lower airway secretions of individuals with asthma compared to that in healthy individuals (52, 53). Enrichment of *Proteobacteria* has also been identified in the lungs of chronic obstructive pulmonary disease (COPD) patients compared with those of healthy smokers and nonsmokers (54, 55). In addition, in a recent Finnish study, increased numbers of *Proteobacteria* in the intestine as a result of antibiotic use have been shown to be associated with an increased asthma risk in 2- to 7-year-old children (56). Combined analysis with heat map data revealed that the dramatic increase in *Proteobacteria* was mainly attributed to *Escherichia-Shigella* at the genus level. Although shigellosis in humans is a disease of the intestine and the roles of *Shigella* in lung diseases have not been documented in humans, it does infect the lungs and does cause disease in mouse models (57, 58). The roles of *Bacteroidetes* in lung diseases probably depend on the exact disease. A higher intestinal *Bacteroidetes* abundance was found to be associated with an increased asthma risk in 2- to 7-year-old children (56); however, a markedly lower abundance of gut *Bacteroidetes* was detected in digestive tract samples from cystic fibrosis (CF) patients than in samples from non-CF patients (59). Interestingly, although the abundance of the *Firmicutes* decreased significantly, there was a remarkable increase in the *Lactobacillus* abundance at the genus level (see Fig. S1B in the supplemental material). This could be a result of the expansion of antibiotic-resistant strains during the course of antibiotic treatment. Indeed, a marked enrichment of *Lactobacillus* was observed in the lung tissue of COPD patients compared with that of nonsmokers, smokers without COPD, and patients with CF (60). An increased representation of *Lactobacillus* has also been reported in a lipopolysaccharide/elastase-challenged mouse model of COPD (61).

Taken together, our study underscores the importance of the host microbiota in modulating host immune responses to respiratory infections and supports the possibility of controlling the severity of B. pertussis infections in humans by manipulating the host microbiota. Our study also provides novel insights into our understanding of B. pertussis pathogenesis. Future work should be focused on deciphering the exact cellular and molecular mechanisms by which the host microbiota modulates CD4^+^ T cell generation and the expression of PD-1 on T cells in response to B. pertussis infection. The further characterization and isolation of specific bacteria that are protective or that play key roles in B. pertussis pathogenesis are also of great importance and warrant further investigation.

## MATERIALS AND METHODS

### Animals.

Four- to 5-week-old female BALB/c mice were obtained from the Animal Center of Slaccas (Shanghai, China). The mice were kept under specific-pathogen-free (SPF) conditions in individual ventilated cages (IVCs). This study was carried out in accordance with the recommendations of the *Guide for the Care and Use of Laboratory Animals* (62). The protocol was approved by the Bioethics Committee of Fudan University.

### Bacterial strains.

The B. pertussis strain used in this study, BPMM, is a streptomycin-resistant derivative of strain ATCC 18323 (ATCC 9797; kindly provided by the Chinese Center for Disease Control and Prevention, China). BPMM was grown on Bordet-Gengou (BG) agar (Difco Laboratories, Detroit, MI, USA) supplemented with 1% glycerol, 20% defibrinated sheep blood, and 100 μg/ml streptomycin for 4 days at 37°C.

### Antibiotic treatment.

Mice were treated for 3 weeks with a cocktail of ampicillin (1 g/liter), vancomycin (500 mg/liter), neomycin sulfate (1 g/liter), and metronidazole (1 g/liter) in drinking water as previously described (21, 26, 63). The antibiotic-containing water was changed once a week.

### Intranasal inoculation.

B. pertussis infection was carried out 3 days after the cessation of antibiotic treatment. Briefly, 5 × 10^6^ CFU of BPMM bacteria in 20 μl sterile phosphate-buffered saline (PBS) supplemented with 0.05% Tween 80 (Sigma) (PBST) was administered to sedated mice intranasally (i.n.) once or twice as previously described (64). For mice infected with B. pertussis twice, the first infection was as described above and the second infection was given 4 weeks later at the same dosage. Blood and spleens were collected 2 weeks after the second infection.

### Lung colonization profiles.

Four adult female BALB/c mice were i.n. administered 5 × 10^6^ CFU of BPMM in 20 μl. At the time points indicated above, four animals per group and per time point were euthanized; their lungs were individually harvested and homogenized as described previously (65).

### Microbiota analyses.

Fresh stool pellets were obtained just before the mice were euthanized. After euthanasia, lung tissues were collected. The collected samples were immediately frozen and stored in empty Eppendorf tubes at −80°C. DNA was extracted from the feces using a QIAamp Fast DNA stool minikit (Qiagen) according to the manufacturer’s instructions. DNA was extracted from the lungs using a QIAamp DNA minikit (Qiagen). The bacterial 16S rRNA gene V4 region was amplified by PCR using primers 515F (5′-GTGCCAGCMGCCGCGGTAA-3′) and 806R (5′-GGACTACHVGGGTWTCTAAT-3′). Sequencing was performed on an Illumina HiSeq2500 platform (Illumina) per the manufacturer’s guidelines. The quality of the sequence was controlled with the Qiime script (v1.7.0; http://qiime.org/scripts/split_libraries_fastq.html) and clustered into operational taxonomic units (OTUs) using Uparse software (v7.0.1001; Uparse). For each experiment and sequencing run, a shared community file and a phylotyped file were generated using OTUs binned at 97% identity via the Mothur program against the SILVA small-subunit rRNA database (http://www.arb-silva.de/). OTU-based alpha and beta diversity was analyzed using Qiime software (v1.7.0).

### Antibody detection.

Blood was collected from infected or control mice by the use of retro-orbital bleeds, and serum was stored at −80°C for subsequent use in the experiments. The presence of antibodies in the serum was measured by ELISA. Ninety-six-well microtiter plates (Costar; Corning) were coated overnight at 4°C with 100 μl of 0.1 M carbonate buffer (pH 9.6) containing 2 μg/ml of BPMM whole-cell lysate. After blocking with 2% bovine serum albumin (BSA) in PBS containing 0.1% Tween 20, 100 μl of serially diluted serum was added to the wells. The plates were incubated at 37°C for 1.5 h, rinsed in PBS–0.1% Tween 20, and incubated at 37°C for 1 h with 100 μl of appropriately diluted horseradish peroxidase (HRP)-conjugated goat anti-mouse IgG (H+L; Sigma), HRP-conjugated goat anti-mouse IgG1 and IgG2a (Abcam), and HRP-conjugated goat anti-mouse IgM and IgA (Bio-Rad) secondary antibodies. The reaction was then developed with 100 μl of tetramethylbenzidine liquid substrate solution (catalog number T4444; Sigma) at room temperature for 30 min in the dark and stopped by the addition of 1 M sulfuric acid. The absorbance at 450 nm was measured by an ELISA plate reader (Tecan Sunrise).

### Passive transfer experiment.

High-titer antipertussis immune sera were generated in 20 adult BALB/c mice nasally infected twice at a 4-week interval with live B. pertussis bacteria. The immune sera from each mouse group were collected 2 weeks after the boost and pooled. The antipertussis antibody titer was measured by ELISA. The immune sera were filter sterilized, heat treated at 56°C for 30 min, and stored at −80°C until further use. Sera from naive control mice were also collected. Four- to 5-week-old mice either were treated for 3 weeks with the antibiotic cocktail in drinking water as described above or remained untreated. B. pertussis infection was carried out 3 days after the cessation of antibiotic treatment. One day before intranasal B. pertussis infection, antibiotic-treated mice and naive mice were i.p. injected with 200 μl of naive or anti-BPZE1 immune serum. Intranasal B. pertussis infection was carried out as described above. Lung colonization of B. pertussis was measured at 4 h, 3 days, and 7 days after infection and compared.

### Flow cytometric analysis.

Spleens from individual mice were harvested, and single-cell suspensions were prepared by meshing the mouse spleens through a 70-μm-mesh-size cell suspension mesh (BD), followed by centrifugation on Ficoll-Paque Plus medium (GE Healthcare) for 20 min at 600 × *g* at room temperature. Cells were collected and washed once with sterile fluorescence-activated cell sorting buffer (2% fetal calf serum [FCS] and 5 mM EDTA in PBS). Splenocytes (10^6^) were stained with the following antibodies: LIVE/DEAD fixable viability dye (Thermo Fisher), anti-mouse CD4-fluorescein isothiocyanate, anti-mouse CXCR5-phycoerythrin (PE), anti-mouse CD279-allophycocyanin (APC), anti-mouse TCRβ chain-BV510, anti-mouse CD19-APC, anti-mouse CD38-peridinin chlorophyll protein-Cy5.5, anti-mouse CD95-PE-Cy7, or isotype control antibodies. All antibodies were purchased from BD Pharmingen. Data were collected on a BD FACSAria flow cytometer (BD Biosciences) and analyzed with FlowJo software (TreeStar Inc., Ashland, OR, USA). A total of 50,000 cells were collected for each sample.

### PC ELISPOT assay.

The frequency of antigen-specific antibody-secreting plasma cells (PCs) was determined by an ELISPOT assay using a mouse ELISPOT set (BD Pharmingen) according to the manufacturer’s instructions. Briefly, 96-well microplates (Millipore, Bedford, MA) were precoated with 100 μl of 10-μg/ml BPMM whole-cell lysate in sterile PBS overnight at 4°C, washed three times, and blocked for 2 h at room temperature with RPMI 1640 containing 10% FCS. Single-cell suspensions of individual spleens from naive, B. pertussis-infected, and antibiotic-treated, B. pertussis-infected mice were then plated onto the plates and incubated overnight at 37°C in a 5% CO_2_ atmosphere. The plates were then washed, followed by addition of biotin-conjugated rat anti-mouse Ig, IgG2a, and IgG1 (all antibodies were from BD Biosciences) for 2 h at room temperature. For the detection of antigen-specific IgG, HRP-conjugated goat anti-mouse IgG (Sigma) was used directly. After washing, streptavidin-HRP conjugate was added and the mixture was incubated at room temperature for 1 h. The wells were washed again and developed with a 3-amino-9-ethylcarbazole (AEC) substrate solution until spots were visible. After drying, spot-forming cell numbers were counted by use of a Bioreader 4000 reader (Applied Biosystems). Six animals per group were assayed individually.

### Statistical analysis.

Quantitative variables were expressed as means ± standard deviations (SD). Comparisons between two groups were performed by Student's *t* test. Data with an abnormal distribution were analyzed by the Mann-Whitney test. A *P* value of less than 0.05 was considered significant.

## Supplementary Material

Supplemental file 1
