# Supplementary material for: Commensal Microbes Affect Host Humoral Immunity to Bordetella pertussis Infection
Source: Infect Immun. 2019 Sep 19;87(10):e00421-19. doi: 10.1128/IAI.00421-19 (PMC6759300; doi:10.1128/IAI.00421-19)
Supplement: Supplemental file 1 [file IAI.00421-19-s0001.pdf]

**SUPPLEMENTARY FIGURES**

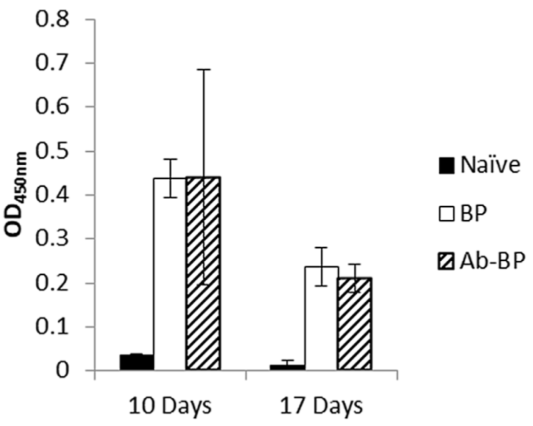


**FIGURE S1** Systemic anti-*B. pertussis* IgM antibody in antibiotic-treated mice and non-treated naïve mice 10 and 17 days after *B. pertussis* infection. Sera were diluted 1:400 and 1:1000 at 10 and 17 days post infection, respectively. Four mice per time point were individually analyzed. Data are representative of two independent experiments.

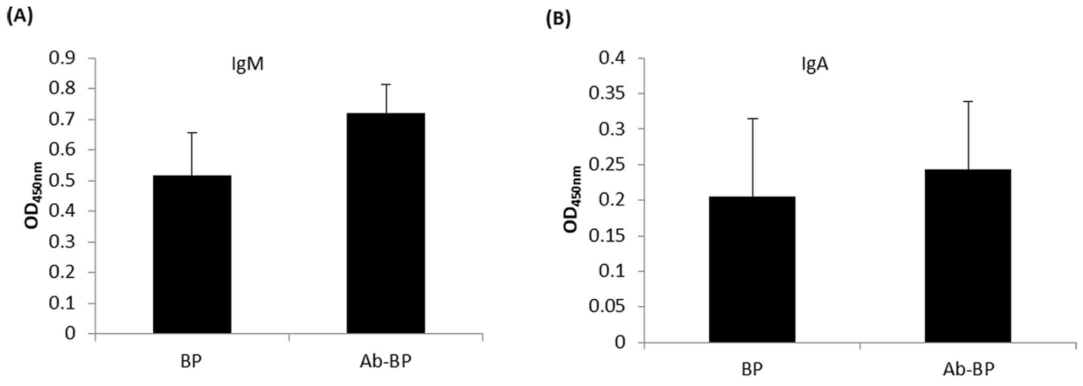


**FIGURE S2** Systemic anti-*B. pertussis* IgA (A) and IgM (B) antibody in antibiotic-treated mice and non-treated naïve mice 14 days after secondary *B.*

*pertussis* infection. Sera were diluted 1:4000 and 1:8000 at 10 and 17 days post infection, respectively. Four mice per time point were individually analyzed. Data are representative of two independent experiments.

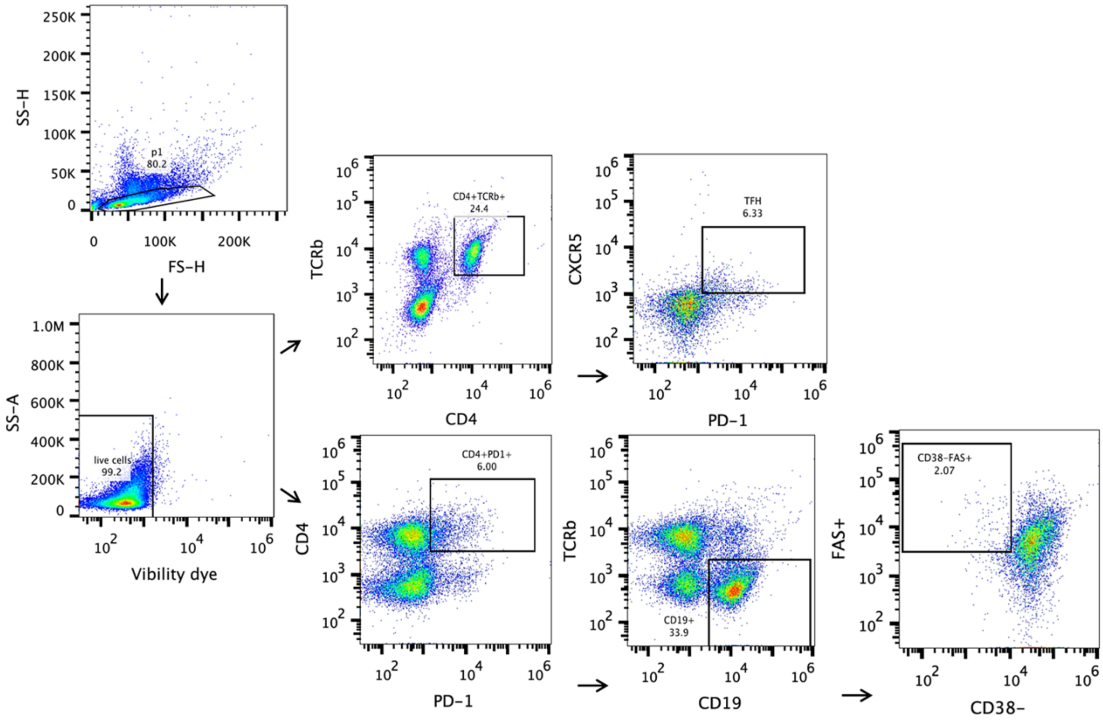


**FIGURE S3** Stepwise flow cytometric gating strategy for the identification of CD4 T cells, GC B cells, T$_{fh}$ cells and PD-1 expression on CD4+ T cells. Splenocytes (10$^{6)}$ were stained with the following antibodies: LIVE/DEAD fixable viability dye (Thermo Fisher), Anti-Mouse CD4-FITC, Anti-Mouse CXCR5-PE, Anti-Mouse CD279-APC, Anti-Mouse TCRβ chain-BV510, anti-mouse CD19-APC, anti-mouse CD38-PerCP-cy$^{TM}$5.5, anti-mouse CD95-PE-cy$^{TM}$7 or isotype control antibodies. Viable lymphocytes (P1) were fist selected based on FS/SS and LIVE/DEAD viability

dye. Tfh and GC B cells were identified in live lymphocytes (P1) by CD4+TCRβ+PD-1+CXCR5+ and CD19+CD38-Fas+, respectively. The frequencies of different cell populations were expressed as percentage within viable lymphocytes (P1).
